# Data‐Driven Placement of PM_2.5_ Air Quality Sensors in the United States: An Approach to Target Urban Environmental Injustice

**DOI:** 10.1029/2023GH000834

**Published:** 2023-09-13

**Authors:** Makoto M. Kelp, Timothy C. Fargiano, Samuel Lin, Tianjia Liu, Jay R. Turner, J. Nathan Kutz, Loretta J. Mickley

**Affiliations:** ^1^ Department of Earth and Planetary Sciences Harvard University Cambridge MA USA; ^2^ Center for the Environment Harvard University Cambridge MA USA; ^3^ Department of Computer Science Harvard University Cambridge MA USA; ^4^ Department of Earth System Science University of California, Irvine Irvine CA USA; ^5^ Department of Energy Environmental and Chemical Engineering Washington University St. Louis MO USA; ^6^ Department of Applied Mathematics University of Washington Seattle WA USA; ^7^ John A. Paulson School of Engineering and Applied Sciences Harvard University Cambridge MA USA

**Keywords:** fine particulate matter (PM_2.5_), sensor placement, sensor networks, environmental justice, citizen science

## Abstract

In the United States, citizens and policymakers heavily rely upon Environmental Protection Agency mandated regulatory networks to monitor air pollution; increasingly they also depend on low‐cost sensor networks to supplement spatial gaps in regulatory monitor networks coverage. Although these regulatory and low‐cost networks in tandem provide enhanced spatiotemporal coverage in urban areas, low‐cost sensors are located often in higher income, predominantly White areas. Such disparity in coverage may exacerbate existing inequalities and impact the ability of different communities to respond to the threat of air pollution. Here we present a study using cost‐constrained multiresolution dynamic mode decomposition (mrDMDcc) to identify the optimal and equitable placement of fine particulate matter (PM_2.5_) sensors in four U.S. cities with histories of racial or income segregation: St. Louis, Houston, Boston, and Buffalo. This novel approach incorporates the variation of PM_2.5_ on timescales ranging from 1 day to over a decade to capture air pollution variability. We also introduce a cost function into the sensor placement optimization that represents the balance between our objectives of capturing PM_2.5_ extremes and increasing pollution monitoring in low‐income and nonwhite areas. We find that the mrDMDcc algorithm places a greater number of sensors in historically low‐income and nonwhite neighborhoods with known environmental pollution problems compared to networks using PM_2.5_ information alone. Our work provides a roadmap for the creation of equitable sensor networks in U.S. cities and offers a guide for democratizing air pollution data through increasing spatial coverage of low‐cost sensors in less privileged communities.

## Key Points

- A method using pollution data to diagnose the optimal and equitable placement of PM_2.5_ sensors is presented
- By incorporating race and income information, the method shifts the sensor distribution to more nonwhite and low‐income neighborhoods
- The approach provides a roadmap for urban areas to create low‐cost sensor networks that address America's legacy of environmental racism

## 1 Introduction

Fine particulate matter (PM_2.5_) air pollution poses the greatest environmental risk to public health (Fann et al., 2012; Murray et al., 2020). However, the deployment of a distributed sensor network to monitor PM_2.5_ pollution is financially and resource intensive. Interpreting measurements of PM_2.5_ is particularly difficult due to its many outdoor sources (e.g., wildfires or vehicular combustion) and its sensitivity to meteorological conditions (Bond et al., 2007; McDuffie et al., 2021; Tai et al., 2012). In addition, the relatively short atmospheric lifetime of PM_2.5_ leads to large spatial and temporal variation of PM_2.5_ exposure, even at the neighborhood scale (Clark et al., 2011; Dai et al., 2020; Liu et al., 2009). In the United States, communities of color are disproportionately exposed to higher levels of PM_2.5_ air pollution at all income levels (Lane et al., 2022; Tessum et al., 2021). Moreover, low‐cost sensor network initiatives have led to sensors being systematically deployed in wealthier, predominantly White neighborhoods (deSouza & Kinney, 2021). Here we provide an intentional sensor network optimization framework that takes into account the multiscale variability of PM_2.5_ and two socioeconomic metrics (race and income) to determine the optimal and equitable placement of PM_2.5_ monitors.

In the United States, monitoring of PM_2.5_ has long relied on networks owned and operated by state, local, and tribal agencies using regulatory grade (Federal Reference Method and Equivalent Method) samplers and monitors. The Environmental Protection Agency (EPA) has promulgated PM_2.5_ minimum monitoring network requirements, based on Metropolitan Statistical Area (MSA) population, and additional design criteria for selecting monitoring locations (Code of Federal Regulations, 2023). These state or local air monitoring stations, hereafter called the “EPA monitors” are deployed primarily to determine compliance with the National Ambient Air Quality Standard yet have ancillary purposes such as providing data to assess air pollution impacts on public health. These sites were originally intended to monitor high‐emitting point sources (e.g., coal‐fired power plants, on‐road traffic) and air pollution in areas with relatively high population. As a result, EPA monitors are not equally distributed across the United States and are not designed to sample the full range of pollutant concentrations (Di et al., 2019; Kelp et al., 2022; Marlier et al., 2022). In part due to the high cost to purchase and maintain regulatory grade monitoring infrastructure, new monitoring sites are added infrequently (US EPA, 2020), and the EPA is considering incorporating networks of low‐cost and crowdsourced sensors to supplement the current EPA network (Barkjohn et al., 2021).

The rise of low‐cost, crowdsourced sensor networks has greatly increased the spatiotemporal monitoring of PM_2.5_ monitoring in the United States. Low‐cost sensors can report measurements publicly in real‐time (Snyder et al., 2013), and recent studies have focused on their calibration (Barkjohn et al., 2021; Delp & Singer, 2020; deSouza et al., 2022), ability to capture wildfire smoke in the wildland‐urban interface (Burke et al., 2022; Holder et al., 2020; Kramer et al., 2023), and skill in characterizing indoor PM_2.5_ from outdoor pollution (Liang et al., 2021; May et al., 2021). While low‐cost sensor networks monitoring PM_2.5_ in urban areas are available, these networks are largely designed by volunteers or are focused on points of interest (e.g., hospitals, elderly care homes, or bus stops) (Esie et al., 2022; Mousavi et al., 2021; Sun et al., 2019). Such networks do not consider the spatial features or temporal dynamics of air pollution, and only recently have these factors been considered when designing locally‐deployed sensor networks (Frederickson et al., 2022). The PurpleAir network is currently the most extensive crowdsourced PM_2.5_ sensor network in the world, with over 10,000 devices globally and over 2,000 outdoor sensors in the United States (Barkjohn et al., 2021).

While device manufacturers such as PurpleAir (with its allied data platform) and organizations such as OpenAQ (with its open‐source air quality data platform) promote the democratization of air pollution monitoring data, the selection of these sensor locations suffers from systematic racial and income biases. The crowdsourced networks often rely on volunteers who are responsible for the installation and upkeep of each sensor, resulting in deployment in predominantly White areas characterized by higher incomes and levels of education relative to US census tracts with EPA monitors (deSouza & Kinney, 2021). In addition, areas with a higher density of low‐cost sensors tend to report lower annual‐average PM_2.5_ concentrations than the EPA monitors in all states except California (deSouza & Kinney, 2021). While well‐intentioned, these citizen science efforts can exacerbate disparities in the spatial coverage of PM_2.5_ monitors, limit the pursuit of environmental justice, and may further perpetuate inequality in PM_2.5_ monitoring (Sorensen et al., 2019; Tubridy et al., 2022). Such considerations are especially pressing given the EPA's American Rescue Plan Enhanced Air Quality Monitoring for Communities, an initiative which provides funding to enhance ambient air quality monitoring in and near underserved communities across the United States (US EPA, 2022).

A multitude of studies describe disparities in exposure to air pollution among racial minorities and people of low socioeconomic status (SES) in the United States, but few propose frameworks to start addressing these inequalities (Fann et al., 2011; Gardner‐Frolick et al., 2022; Van Horne et al., 2022; Wang et al., 2022). A recent study highlights the historical discriminatory practice of redlining, in which services such as mortgages, insurance loans, and other financial services are systematically denied to residents of certain areas based on their race or ethnicity, and reveals how this practice continues to shape systemic disparities in air pollution exposure in the United States (Lane et al., 2022). Racial minorities and low SES groups are at a higher risk of death and disease from PM_2.5_ exposure (Bell & Ebisu, 2012; Jbaily et al., 2022; Liu et al., 2021; Mikati et al., 2018; Miranda et al., 2011). While absolute disparities in air pollution in the US have declined significantly since 2000, relative disparities between White and minority groups persist (Clark et al., 2014; Colmer et al., 2020). Past studies have comprehensively investigated racial/ethnic disparities in air pollution exposure; such studies have relied on ground‐based monitoring data (Clark et al., 2014; Demetillo et al., 2020, 2021; Jbaily et al., 2022; Kerr et al., 2021; Liu et al., 2021; Tessum et al., 2021). Given the heightened risk of racial minorities and low SES groups to PM_2.5_ pollution in the United States, a PM_2.5_ sensor network biased toward wealthier or whiter communities may lead to mischaracterizations of exposures and inaccurate assessment of the health impacts of such pollution. Proposing an equitable PM_2.5_ monitoring network presents a beneficial first step in starting to address this inequality issue.

In this study, we demonstrate a data‐driven approach that determines the optimal and equitable placement of sensors to capture PM_2.5_ concentrations and variability, while considering socioeconomic metrics (race, income) across four urban areas with histories of segregation in the United States. We use multiresolution dynamic mode decomposition with cost constraints (mrDMDcc), which recursively decomposes a data set into low‐rank spatial modes and their temporal Fourier dynamics, while incorporating a socioeconomic cost function in the optimization (Clark et al., 2019; Kutz et al., 2016; Manohar et al., 2019; Proctor et al., 2014). This algorithm allows for the creation of a library of modes that not only captures PM_2.5_ concentrations spatially and temporally on short (weekly) and long‐term (years to decade) timescales, but also incorporates cost‐constraining functions that optimize sensor placement based on race and income metrics relevant to environmental justice. Our algorithm can capture a finer level of spatial and temporal variability in a data set that would otherwise be averaged out using traditional mean or maximum PM_2.5_ metrics. We previously applied a similar algorithm, but without the socioeconomic cost function, to identify the optimal placement of PM_2.5_ sensors across the contiguous United States (Kelp et al., 2022). Here we extend that work by designing optimal PM_2.5_ sensor networks that account for race and income in four cities: St. Louis, MO; Houston, TX; Buffalo, NY; and Boston, MA. We identify monitoring locations on the spatial scale of 1 km^2^, which is in accords with neighborhood scale of representation for PM_2.5_ monitoring siting. We compare our results to the current networks of EPA and PurpleAir monitors (acknowledging that there may be other low‐cost sensor networks in one or more of these cities), and we contrast the mrDMDcc network designed with only air pollution modal information against those with race and with income included in the optimization.

## 2 Methods

### 2.1 PM_2.5_ Data Set

We use a data set consisting of modeled daily PM_2.5_ concentrations at 1 km × 1 km for the contiguous United States for the period January 2000 to December 2016. This data set was produced through a data fusion method using ensemble machine learning to combine surface monitoring measurements, satellite aerosol optical depth, land‐use data, and chemical transport model results, among other variables (Di et al., 2019, 2021). We subset the data set to encompass each city described in Section 2.3. While this PM_2.5_ data set tends to underpredict PM_2.5_ on high‐pollution wildfire days in the Western United States (Considine et al., 2022), these underestimates are not a concern for our analysis as we focus on urban areas in the central and eastern United States. We remove missing values from the data set by applying a mask to the spatial grid.

### 2.2 Census Data

For our racial and economic analysis of the urban areas, we obtain sociodemographic features from the 2020 American Community Survey using the R package tidycensus. We use socioeconomic data at the census tract level and interpolate these features to the Di et al. (2021) grid to get census estimates at the centroids of the 1 km × 1 km grid cells. For each grid cell in an urban area, we calculate the proportion of nonwhite individuals in terms of population and annual median income. We define the nonwhite proportion as one minus the non‐Hispanic White proportion.

### 2.3 Urban Areas

**Figure 1:**
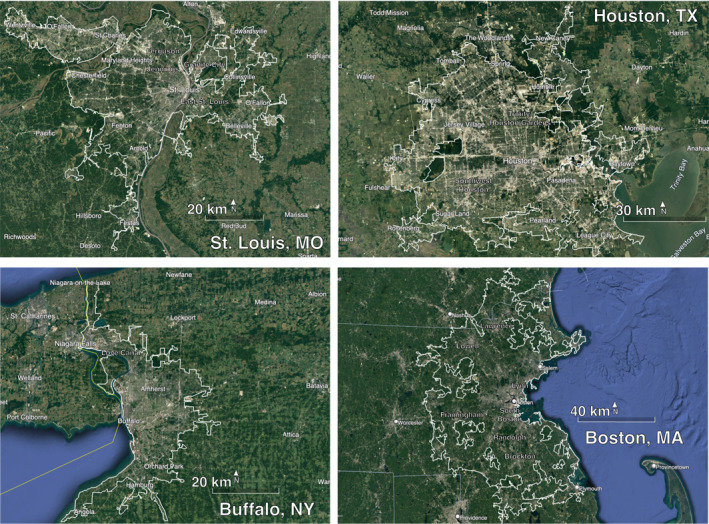
Spatial extent of St. Louis, Houston, Buffalo, and Boston metropolitan areas. Image source: Google Earth Engine software with data from SIO, NOAA, U.S. Navy, NGA, GEBCO, and images from Landsat/Copernicus, U.S. Geological Survey.

We focus our analysis on four urban areas in the United States with high rates of racial segregation and underserved PM_2.5_ monitoring networks: St. Louis, MO, Houston, TX; Buffalo, NY; and Boston, MA. Although many cities in the United States are racially segregated, we select these urban areas based on their geographic diversity and long histories of environmental injustice. Figure 1 shows the locations of these urban areas with geographic landmarks. The extent of the urban area domain is specified by the U.S. Census Bureau Shapefile for Urban Areas (https://www.census.gov).

**Figure 2:**
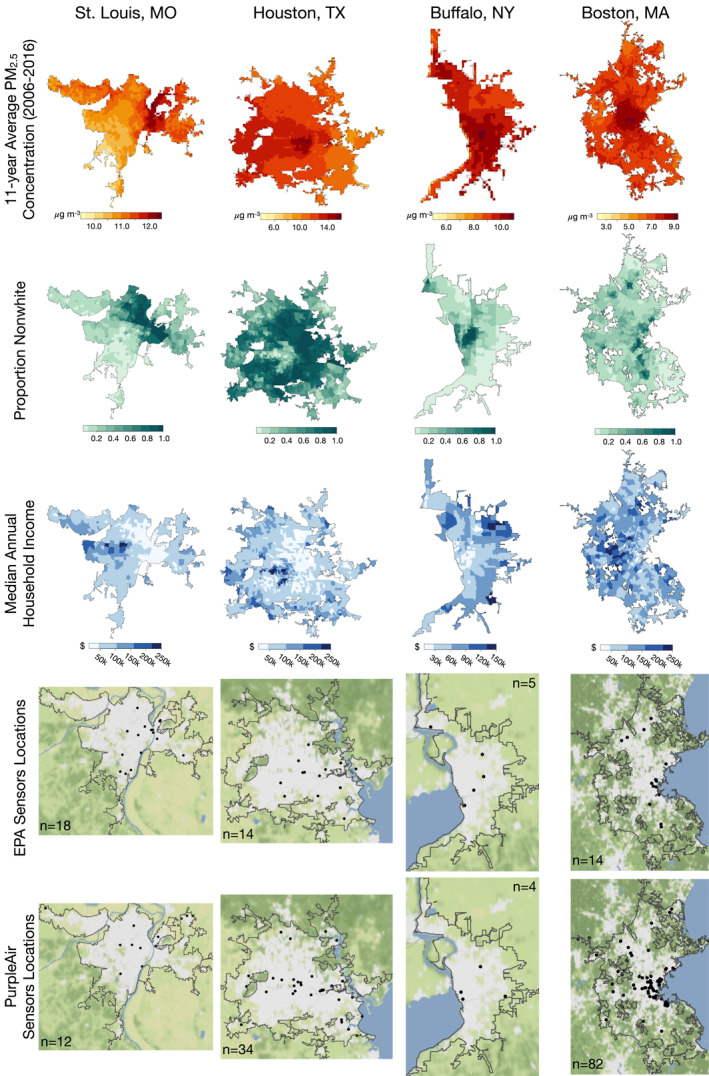
Maps of decadal PM_2.5_ concentrations, socioeconomic inequality metrics, and EPA and PurpleAir sensor locations for St. Louis, Houston, Buffalo, and Boston metropolitan areas. The 11‐year mean of annual averages of PM_2.5_ over 2006–2016 are from estimates from Di et al. (2021). The proportion nonwhite and median annual household income are from the 2020 American Community Survey interpolated onto the centroids of the Di et al. (2021) PM_2.5_ data set. EPA (locations used in Di et al. (2021)) and PurpleAir (downloaded on 19 July 2021) sensor locations are gridded onto the same 1 km × 1 km Di et al. (2021) grid. White areas of the sensor location maps represent the built environment, while the shades of green represent the natural vegetation colors of the area.

In all urban areas, decadal PM_2.5_ concentrations are generally higher in more nonwhite and lower income neighborhoods (Figure 2). St. Louis (population: 2.8 million, area: 22,000 km^2^) displays stark segregation, with large nonwhite populations found north of downtown in majority‐Black neighborhoods such as Jennings and Ferguson in Missouri and East St. Louis in Illinois. These neighborhoods in greater St. Louis have long histories of being targeted by structurally racist polices, which have shaped the city landscape (Johnson, 2020). Houston (population: 7.2 million, area: 26,000 km_2_) has one of the largest nonwhite Hispanic populations in the United States and is home to nearly a quarter of the nation's chemical refineries. Most of these petrochemical refineries are located near the Houston Ship Channel along the Buffalo Bayou River between Baytown and Downtown Houston, an area containing a disproportionate number of low‐income and nonwhite households (Demetillo et al., 2020; Jang et al., 2021; Sansom et al., 2017). Buffalo (population: 1.1 million, area: 4,000 km^2^) has large populations of nonwhite residents in the areas north and east of downtown. The Buffalo metropolitan area has a long history of environmentally racist policies, including toxic chemical dumping in predominantly nonwhite neighborhoods such as Niagara Falls and Love Canal in the 1970s (Fletcher, 2021). Nonwhite communities continue to disproportionately experience exposure to air pollution from major roadways and have limited access to environmental amenities like parks and other public spaces (Drake et al., 2022; Krieg, 2005; Phillips et al., 2007). Finally, Boston's (population: 4.9 million, area: 12,000 km^2^) modern demographics are influenced by its early 20th century redlining policies, with large nonwhite populations found in the residential suburbs of South Boston, Lowell, and Lawrence among others (Harvard Chan‐NIEHS Center for Environmental Health, 2021).

In our selected urban areas, the number and locations of EPA monitors are sparse, and the locations of PurpleAir monitors are generally in whiter and higher‐income census tracts, as first indicated by deSouza et al. (2022) (Figure 2). On average, EPA monitors are in lower income and higher proportion nonwhite areas (St. Louis: 47% nonwhite, $66,000 median income; Houston: 79%, $55,000; Buffalo: 31%, $44,000; Boston: 52%, $83,000) than PurpleAir monitors (St. Louis: 45% nonwhite, $60,000 median income; Houston: 59%, $81,000; Buffalo: 36%, $67,000; Boston: 41%, $98,000). The St. Louis metropolitan area has the most EPA monitors out of the four cities, with 18, but only 12 PurpleAir monitors. Buffalo has the fewest EPA and PurpleAir monitors out of the four cities, with five and four, respectively. Boston has the most PurpleAir monitors out of the four cities, with 82.

### 2.4 Sensor Placement Using Multiresolution Dynamic Mode Decomposition With Environmental Justice Cost‐Constraint (mrDMDcc)

We use multiresolution dynamic mode decomposition (mrDMD), which recursively decomposes a data set into low‐rank spatial modes and their temporal Fourier dynamics (Kutz et al., 2016; Manohar et al., 2019). mrDMD has been shown to capture PM_2.5_ concentrations spatially and temporally on short (daily) and long‐term (years to decade) timescales, and to incorporate information from transient phenomena, such as wildfires and temperature inversions, that would otherwise be discarded or averaged out using similar data reduction techniques (Kelp et al., 2022). The algorithm can thus capture a finer level of spatial and temporal variability in a data set that would otherwise be averaged out using traditional mean PM_2.5_ or maximum PM_2.5_ metrics. mrDMD is a dimensionality reduction algorithm, similar to principal components analysis (PCA), but mrDMD is more precise in capturing spatiotemporal variability than methods based on singular value decomposition such as PCA (Manohar et al., 2019). A formal expansion of the DMD and mrDMD theory and modeling approach may be found elsewhere (Kelp et al., 2022; Kutz et al., 2016; Manohar et al., 2019).

The mrDMD algorithm operates by decreasing the time domain by a factor of two at each successive decomposition level. We apply mrDMD to training windows starting at 11.4 years (*M* = 4,096 days), followed by 10 decomposition levels so that the shortest frequency is weekly. This approach thus yields a long‐term mode characterizing the average PM_2.5_ concentrations over 11.4 years, with potential identification of transient pollution events spanning timescales from 5.7 years (2,048 days) to 8 days. Recent studies have demonstrated the sensitivity of PM_2.5_ across the United States to large‐scale, multi‐year meteorological patterns such as El Niño or the Atlantic Multidecadal Oscillation (Previdi & Fiore, 2019; Shen et al., 2017). As we shall see, however, most of the events identified by mrDMD occur on timescales lasting days to weeks. Here, we focus our analysis on the last 11.4 years (4,096 days) of our data set: September 2005–December 2016.

We use the matrix libraries containing all mrDMD modes as tailored basis sets $\psi_{r}\in R^{n\times r}$ to optimize for sensor placement. We identify the optimal sensor locations by employing QR pivoting to our mrDMD basis sets (Heck et al., 1998; Manohar et al., 2019). QR pivoting is a “greedy” selection algorithm that is computationally efficient for finding near‐optimal sensor locations. Greedy approaches are often favored over other optimization techniques, as the true optimal solution often involves a combinatorially intractable optimization. QR column pivoting identifies rows in the modal library *ψ*_*r*_ with the highest 2‐norm, which corresponds to locations with the largest PM_2.5_ modal frequencies and therefore greatest variability. The reduced matrix QR factorization with column pivoting decomposes a matrix $A\in R^{m\times n}$ into a unitary matrix *Q*, an upper‐triangular matrix *R*, and a column permutation matrix *C* ^*T*^, such that AC^*T*^ = QR. Thus, the QR factorization with column pivoting yields *r* point sensors (pivots) that best sample the *r* tailored basis modes *ψ*_*r*_:

(1) $$\psi_{r}^{T}C^{T}=QR$$

That is, each QR pivot identifies those spatial locations in the modal library that exhibit the most variability and where sensor placement would capture the greatest number of significant pollution episodes above background concentrations. To reiterate, mrDMD intends to capture spatial and temporal variability in pollution without taking into account other considerations.

Here, we present an extension to the mrDMD framework that considers cost‐constraining functions (Clark et al., 2019) to optimize sensor placement based on racial and income metrics related to environmental justice. The mrDMDcc algorithm is based on the column pivoted QR algorithm described above, where the pivot column is now chosen to balance (a) the decrease in accuracy of capturing the largest air pollution modal signals with (b) the increase in capturing pollution exposure in communities with either a high proportion nonwhite or low‐income populations. To incorporate the cost of a sensor, we create a factorization *l* which satisfies:

(2) $$l=\max_{i=1,\text{…},n-k}{\left\|C^{k,i}\right\|}_{2}-\gamma \eta_{j_{i+k}^{k}}$$

where *k* is the number of sensors specified by the mrDMD library, *n* is the number of columns in the original PM_2.5_ data matrix, *C* is the column permutation matrix, *η* is a vector that contains the socioeconomic cost function, and *γ* is the cost‐balance coefficient that specifies the degree to which the cost function must be obeyed by applying a penalty term. Once calculated, *l* is used to permute the indices in the mrDMD library to find the pivots that satisfy the cost constraint.

A formal expansion of the sparse sensor placement approach and cost‐constraint pseudocode may be found elsewhere (Brunton & Kutz, 2019; Clark et al., 2019; Kelp et al., 2022; Manohar et al., 2018, 2019).

The cost function used here is a step function that penalizes placing sensors too far from majority nonwhite or low‐income neighborhoods. For the cost function vector, all socioeconomic data is rescaled between 0 and 1. The proportion of nonwhite people in a grid cell ranges from 0 to 1, with 0 representing an all‐white population and 1 representing an all‐nonwhite population. The annual median income of a grid cell is normalized according to the maximum value in the census data ($250,000 for St. Louis, Houston, and Boston; $150,000 for Buffalo) and inverted such that 0 indicates highest income and 1 indicates lowest income (Figure S1 in Supporting Information S1). Values of *γ* range from 0 to 0.5 and we select, by inspection, sensor network results that best balance capturing high‐variability PM_2.5_ locations with more nonwhite or lower income areas. We apply a user‐selected *γ* value that prevents the cost function from placing sensors in highly dense clusters that do not improve coverage of vulnerable neighborhoods (Figure S2 in Supporting Information S1). With a PM_2.5_ data set timescale spanning over 11 years to 1 week, the mrDMD modal library typically identifies more than 800 sensors because the more polluting events on shorter timescales that the sensor network attempts to capture, the more sensors it needs to do so (Figure S3 in Supporting Information S1). However, we choose to display the top 250 sensors in St. Louis, Houston, and Boston, and the top 150 sensors in Buffalo, as these numbers of monitors create a realistic distribution of sensors spanning both the urban core and more suburban/rural areas of each city without losing spatial coverage. Generally, we find that under 100 sensors fails to capture many socioeconomic communities in an urban area, while more than 300 sensors lead to overlapping spatial clustering which may be redundant (Figure S4 in Supporting Information S1). However, adding or subtracting 50 sensors from this total does not meaningfully impact the prioritization of the relevant socioeconomic communities. We choose to display 150 sensors in Buffalo as it is the smallest in area (4,000 km^2^) among all urban cities selected in this study. We find that showing more than 150 sensors becomes less interpretable when comparing differences. Additionally, we selected 250 sensors for St. Louis (22,000 km^2^), Houston (26,000 km^2^), and Boston (12,000 km^2^) as this number is sufficient to cover the domain of the MSA and identify patterns among different mrDMDcc optimizations. We acknowledge that the optimal number of sensors may vary for each city, although we did not optimize that number here.

The mrDMDcc algorithm does not require a new simulation when the target number of sensors is changed. This is in contrast to other algorithms where the network size is specified a priori, such as the location‐allocation approach which is an optimization algorithm built into ArcGIS (Kanaroglou et al., 2005).

## 3 Results and Discussion

### 3.1 Optimal and Equitable Sensor Locations

**Figure 3:**
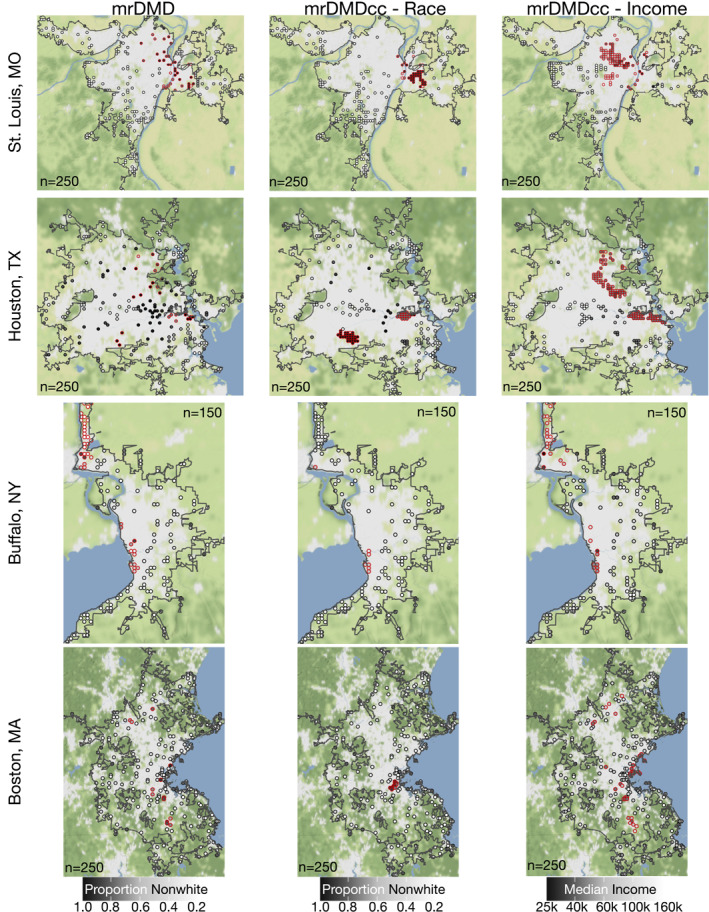
PM_2.5_ sensor locations for St. Louis, Houston, Buffalo, and Boston. Distribution of sensor locations identified as optimal by the mrDMD algorithm, and those identified as optimal and equitable by the mrDMDcc using race and income metrics. All sensor locations are gridded onto the same 1 km × 1 km Di et al. (2021) grid. Dots represent sensor locations with the shading representing the proportion of nonwhite (left and center columns) or low‐income households (right column) in that grid box. Dots outlined in red indicate areas with historic environmental justice issues mentioned in the text—for example, Granite City, IL, and East St. Louis, IL, for the race optimized mrDMDcc case.

In St. Louis, using race and income metrics in the mrDMDcc optimization leads to a greater number of sensors clustered in historically nonwhite and low‐income neighborhoods (Figure 3, top row). We find that the mrDMD algorithm, which takes into account only air pollution modes, distributes sensors throughout the St. Louis urban airshed, with many monitors located some distance away from downtown and the Black northern residential suburbs. This distribution is due to frequently occurring wintertime nitrate events, during which cold temperatures and high‐pressure systems drive nitrate from agricultural activities in the Midwest into particle phase, forming haze (Lee et al., 2006). These haze events result in large PM_2.5_ modal signals above background variability that are located away from the urban city center (Figure S5 in Supporting Information S1). In contrast, the mrDMDcc optimized for race (*γ* = 0.20) shifts more sensors to the historically Black East St. Louis neighborhood. This area not only has the highest proportion of nonwhite residents, but also includes steelworks operations in Granite City, IL, (Lee & Hopke, 2006; Wang et al., 2009) which are a large driver of environmental injustice in the region. The area surrounding Granite City is characterized by high PM_2.5_ variability in our mrDMD modal library (Figure S5 in Supporting Information S1). Finally, the mrDMDcc optimization for income (*γ* = 0.17) shifts more sensors to the lower‐income (and historically Black) northern suburbs of Jennings and Ferguson, communities that have faced many environmental justice issues (Marcantonio et al., 2017). If considering only air pollution modes from the mrDMD sensor network, all these communities would be under monitored.

In Houston, race‐ and income‐optimized mrDMDcc networks shift the distribution of sensors toward the Ship Channel region and majority Black neighborhoods (Figure 3, second row). When considering only air pollution modes, the mrDMD algorithm places sensors throughout the Houston area, with a particular focus around downtown. This distribution is reasonable given that the downtown area typically has the highest concentrations of PM_2.5_ in the region due to industrial sources and heavy‐duty diesel trucking (Demetillo et al., 2020; Du et al., 2019). Evidence of downtown PM_2.5_ emission hotspots and arterial traffic can be seen in the PM_2.5_ modal library (Figure S6 in Supporting Information S1). Here the mrDMDcc optimization that takes into account race information (*γ* = 0.12) shifts more sensors to neighborhoods along the Ship Channel and in majority Black neighborhoods in Southwest Houston. The Ship Channel is a major polluter of the Houston urban core, with epidemiological studies revealing that children living within two miles of the channel are 56% more likely to develop leukemia than the national average (Linder et al., 2008). The race‐optimized sensor locations in Southwest Houston are in a majority Black neighborhood and show PM_2.5_ modal signals suggesting a large presence of on‐road emissions from freeways (Figure S6 in Supporting Information S1) and is upwind from the W.A. Parrish Generating Station, a dual‐fired power plant that includes the largest coal‐fired plant in Texas (Demetillo et al., 2020). Finally, the mrDMDcc optimization with income information (*γ* = 0.13) also shifts sensor placements along the Ship Channel and in the low income (and majority Black) suburbs north of downtown, including Trinity and Houston Gardens. As in St. Louis, we find that mrDMDcc distributes sensors in Houston with a heightened focus on polluted, primarily low‐income, and majority Black neighborhoods that would be relatively under monitored if considering only the air pollution modes.

In Buffalo, all three sensor network optimizations place monitors in the Niagara Falls and Love Canal neighborhoods (Figure 3, third row). Buffalo is the smallest of the urban cities analyzed in this work in terms of both spatial extent and population, and we limit the number of sensors to 150 (Figure S7 in Supporting Information S1). Here we find that the distribution of sensors generated by all three mrDMD approaches yields similar results. The race‐ (*γ* = 0.34) and income‐ (*γ* = 0.32) optimized networks do not shift monitors to the north of downtown Buffalo, where large nonwhite and low‐income populations reside. Although the downtown is represented in the PM_2.5_ modal library (Figure S8 in Supporting Information S1), the largest variability in PM_2.5_ is caused by lake effect meteorology (Spak & Holloway, 2009) and by pollution in the Niagara Falls neighborhoods; both of these effects shift sensor density away from downtown. Nevertheless, the Niagara Falls and Love Canal neighborhoods have known environmental justice pollution issues that affected large nonwhite and low‐income populations (Fletcher, 2002, 2021; Gibbs, 2011; Newman, 2016) that are captured by all mrDMD sensor networks.

In Boston, the income‐optimized mrDMDcc algorithm captures more nonwhite and low‐income neighborhoods than either the race‐optimized algorithm or the mrDMD taking only air pollution modes into account (Figure 3, bottom row). The mrDMD approach places sensors throughout the Boston metropolitan area, with a particular focus on downtown and the area west of Boston. To be sure, the largest emissions of PM_2.5_ are in downtown Boston, which dominate the background variability in the PM_2.5_ modal library and where population density is greatest (Figure S9 in Supporting Information S1). The mrDMDcc race‐optimized algorithm (*γ* = 0.24) shifts more monitors to South Boston, which has the highest proportion of nonwhite residents in this region. However, the large signal from South Boston dominates the mrDMDcc cost function in this case and shifts sensor placement from other nonwhite neighborhoods such as Lowell, Lawrence, and Brockton, causing these locations to lose sensor density. We attribute this result to the mrDMDcc optimization, which prioritizes the largest values in the cost function vector and does not linearly scale other values in importance. On the other hand, the income‐optimized mrDMDcc algorithm (*γ* = 0.10) clusters more sensors in nonwhite neighborhoods since the income metric contains fewer extreme values that can overwhelm the optimization cost function. Using the income‐based sensor optimization, we find more sensors placed in the residential suburbs of Lowell, Lawrence, Brockton, Randolph, and Lynn, which have both major nonwhite and low‐income populations. Designing a distributed sensor network for Boston is challenging in part due to the highly variable meteorological patterns of New England that affect PM_2.5_ concentrations. This variability is caused by New England's physical geography, including its coastal orientation, position within the prevailing westerlies, and presence of mountains, as well as its diverse climate patterns, such as large diurnal changes in temperature, droughts, heavy rainfall, and blizzards (Keim et al., 2005). In addition, the Boston area is predominately White with spatially dispersed nonwhite communities, making it difficult to optimize based on the race metric. Our mrDMD results in Boston thus highlight the importance of considering both race and income in sensor network design due to these computational limitations.

### 3.2 Cumulative Distributions of Sensors

**Figure 4:**
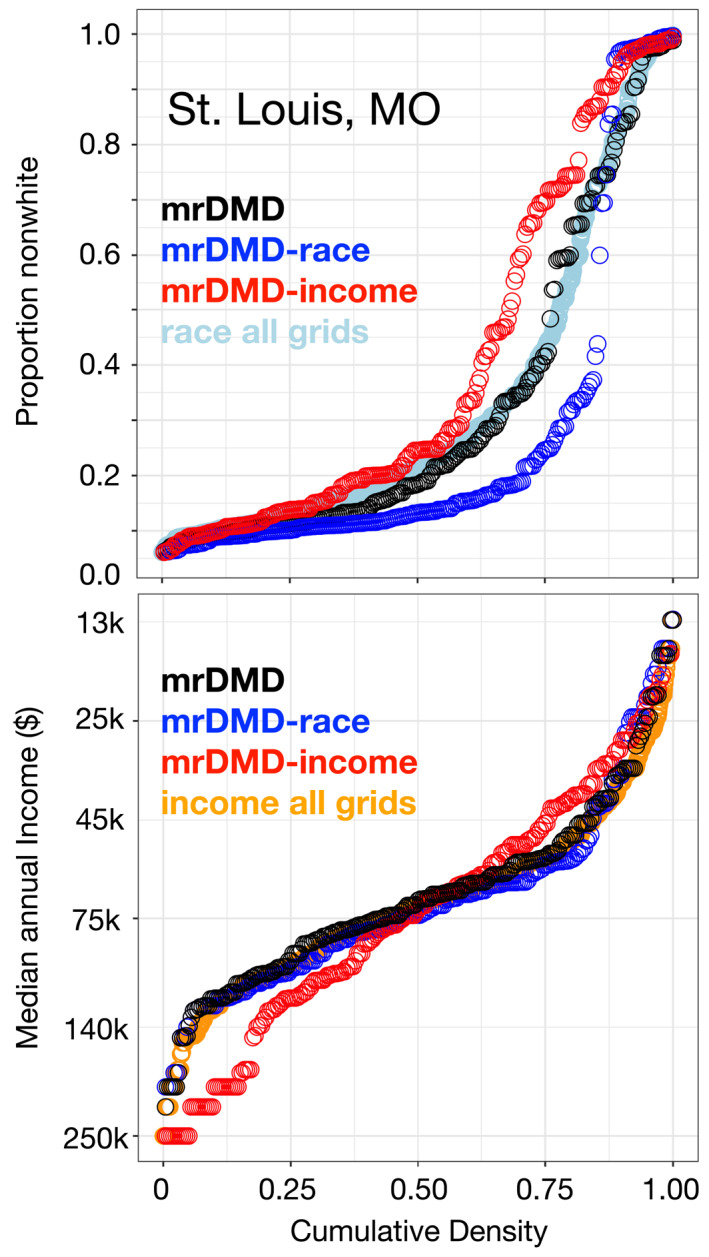
Cumulative frequency distributions for proportion of nonwhite locations and median annual income for the three different sensor network optimizations for St. Louis. Each point represents one sensor location out of the 250 designed for St. Louis. The mrDMD network is designed with only air pollution modal information, mrDMD‐race includes race information from the United States Census in the sensor network optimization, and mrDMD‐income includes annual income information from the Census in the sensor network optimization. An additional set of points in each plot represents the distribution across racial composition (light blue) and income (orange) for a high‐density, uniformly distributed sensor network across all 1 km^2^ grid cells within the city bounds. The *y*‐axis for median annual income has been reversed to make this panel consistent with the other panels, with the neighborhoods of greatest interest in this study plotted at the high end of the distributions.

Cumulative distributions of sensors confirm that mrDMDcc optimizations using environmental justice metrics capture more nonwhite and low‐income neighborhoods than the mrDMD optimization relying on pollution modes alone. Figure 4 shows the cumulative distributions of sensors in St. Louis for proportion of nonwhite residents and median annual income for the three different sensor optimizations methods in this work. The decadal PM_2.5_ concentrations are similar among the three sensor optimizations, with differences of at most 0.25 μg m^−3^ for a given cumulative frequency. This indicates that the methods similarly capture the distribution and extent of decadal mean PM_2.5_ in a metropolitan area, even though daily or weekly PM_2.5_ concentrations may vary substantially across the city. Comparing mrDMD against the distributions for racial composition (Figure 4, light blue) and income (Figure 4, orange) across all 1 km^2^ grid cells within the St. Louis city bounds indicates that the mrDMD method is comparable to random sampling for both socioeconomic and racial metrics. In contrast, the cumulative distributions for the proportion of nonwhite residents suggest that the race‐optimized network captures a relatively higher density of the greatly nonwhite locations.

Figure 4 demonstrates that the mrDMDcc cost function is particularly sensitive to the maximum and minimum values of the environmental justice metrics we supply. The mrDMDcc distributions reflect this sensitivity to the cost function: East St. Louis has the highest values of the proportion nonwhite residents (greater than 95% nonwhite, Figure 2) while Jennings and Ferguson have the lowest annual income values (less than $30,000, Figure 2). As a result, these neighborhoods dominate the race and income optimizations, respectively, and subsequently shift sensor density to these areas (Figure 3). In the middle quartile range of cumulative sensor frequency (e.g., 0.25–0.75), the race‐optimized network captures mostly White neighborhoods. Given the strong racial segregation in St. Louis, relatively few monitors are placed in neighborhoods with 0.3–0.8 fraction of nonwhite populations. At the low end of the race‐optimized distribution are mostly White (and often more rural) communities that have large variability in PM_2.5_ concentrations due to meteorology and regional‐scale haze events (winter nitrate, summer sulfate) rather than from emissions in the urban core of a city. The income‐optimized network, on the other hand, captures more low‐income neighborhoods compared to both the mrDMD and mrDMDcc race‐optimized networks.

Optimizing by income in Houston, Buffalo, and Boston leads to more low‐income and more nonwhite sensor locations compared to both the mrDMD and mrDMDcc race‐optimized networks (Figures S10–S12 in Supporting Information S1). We generally observe that the income metric landscapes have fewer extreme values, which make the optimization problem more tractable for the mrDMDcc framework. However, the income optimizations in St. Louis, Houston, and Buffalo also place sensors in the wealthiest neighborhoods (top 5% of income distribution). These unexpected sensor configurations are likely due to (a) the mrDMDcc algorithm balancing the accumulated error from selecting low‐income populations, which may not have as much air pollution modal variability, or (b) suburban areas having higher relative PM_2.5_ variability because they have lower concentrations than the urban core together with local meteorology or regional scale events (wintertime nitrate, summertime sulfate) causing larger relative impacts. Further investigation of these issues lies beyond the scope of this study, but would be important to consider for further implementation of this work.

While race and income are often correlated in urban areas (Bhutta et al., 2020), the mrDMDcc algorithm tends to capture more nonwhite communities when using income in the cost function than when using race, especially in St. Louis and Houston (Figure S13 in Supporting Information S1). We hypothesize that this finding is due to extreme levels of racial segregation in these cities. As described above in the case of St. Louis, race metrics exhibit greater disparity, both spatially and in terms of minimum and maximum values, leading to a more challenging optimization. Additionally, the “shape” of segregation (Chodrow, 2017) has an impact on the optimizations presented in this study. In cases where large portions of a city are segregated, such as St. Louis and Houston, sensor placement is relatively straightforward. However, if segregated areas are scattered throughout the urban area, as in the case of Boston, the sensor placement becomes more complex. Binning the United States Census race and income data (from which the environmental justice metrics are derived) into coarser statistical groups, such as combining the 90th percentile of nonwhite residents with the 99th percentile, may improve the sensor optimization. However, we do not do so in this work as it would likely average out the most socioeconomically disparate communities. In any event, the large disparity in race values encountered in this work underscore the legacy of stark segregation exhibited in most major American cities (Chodrow, 2017).

## 4 Conclusions

We present a data‐driven approach to identify the optimal and equitable placement of PM_2.5_ sensors to capture extremes of air pollution in four urban cities (St. Louis, Houston, Buffalo, and Boston), all with legacies of segregation and environmental racism. Previous studies have relied on crowdsourced sensor networks, such as PurpleAir, to fill in the gaps of the EPA monitoring network, but such sensors are mainly concentrated in whiter, higher income neighborhoods. This study is the first to diagnose both the optimal and equitable placement of PM_2.5_ sensors, capturing both PM_2.5_ extremes and PM_2.5_ exposure in majority nonwhite or poor neighborhoods. Our method uses multiresolution dynamic mode decomposition with environmental justice cost constraints (mrDMDcc), an approach that takes into account the variability of PM_2.5_ on timescales ranging from 8 days to over a decade and incorporates race and income data from the 2020 United States Census into the optimization. All mrDMDcc sensor networks are data‐driven and constructed from modal libraries, which capture both the background variability and reoccurring high pollution episodes in PM_2.5_ within an urban area. Comparing mrDMD against the distributions for racial composition and income across all 1 km^2^ grid cells within metropolitan city bounds indicate that the mrDMD method is comparable to random sampling from a dense distribution for both socioeconomic metrics (Figure 4, Figures S10–S12 in Supporting Information S1). Our results show that using mrDMDcc to design an air quality sensor network leads to a higher number of sensors placed in historically low‐income and nonwhite neighborhoods with known environmental pollution problems. For example, the mrDMDcc sensor networks highlight neighborhoods along major polluting areas, such as the Ship Channel in Houston and Granite City, IL, in the St. Louis metropolitan area. Such neighborhoods are considerably under monitored when using an approach without considering race or income in the optimization. In Buffalo, a city with only five EPA monitors and four PurpleAir monitors, the mrDMDcc algorithm creates sensor networks with a heightened focus on the Niagara Falls and Love Canal neighborhoods, both of which have long legacies of environmentally racist policies.

Although the mrDMDcc sensor network shifts more monitors to nonwhite and low‐income locations, we find that the algorithm is sensitive to the range of the socioeconomic data supplied. Even though studies have shown that race is the dominant factor in air pollution exposure disparities (Liu et al., 2021), we find that optimizing sensor location by income more often leads to greater coverage in both low‐income and nonwhite neighborhoods compared to optimizing by race (Figures S10–S12 in Supporting Information S1). The mrDMDcc algorithm tends to capture fewer nonwhite communities when using race in the cost function due to the severe racial segregation, both spatially and in terms of the minimum and maximum values supplied to the cost function. These factors lead to a more challenging optimization (Figure S13 in Supporting Information S1). Binning the United States Census race and income data into coarser statistical groups than those used here could improve the sensor optimization but would likely average out the most socioeconomically disparate communities. The large disparity in race values encountered in this work underscores the legacy of stark segregation that endures in most major American cities.

The mrDMDcc algorithm provides a roadmap for designing optimal and equitable PM_2.5_ sensor networks for segregated cities and regions in the United States. Our results indicate that considering social inequality metrics in the optimization of air quality sensor networks can lead to a more equitable distribution of monitoring resources and provide a more comprehensive understanding of the distribution of air pollution within a city. We produce sensor networks with 250 monitors for St. Louis, Houston, and Boston, and 150 monitors for Buffalo, which may seem impractical given that these areas typically have fewer than 50 EPA and PurpleAir monitors combined. However, our results are intended as a strategy for designing a comprehensive sensor network for these urban areas. Extensions to the mrDMDcc framework could incorporate sensor measurements with different levels of accuracy—i.e., high‐cost and low‐cost sensors (Clark et al., 2020)—or could consider cost‐constraining functions using other metrics such as population density (Kanaroglou et al., 2005), fraction of children or the elderly (Sun et al., 2019), Normalized Difference Vegetation Index for green spaces in urban areas (Larkin et al., 2017), and other land use information (Lu et al., 2021). Additional postprocessing of sensor locations may be necessary to better capture spatial gradients coming into and out of communities of interest and determine the threshold at which adding more sensors will lead to redundant overlap. Furthermore, we may use a combination of metrics such as the covariance between race and income. In future work, a more systematic approach could be developed to determine an appropriate *γ* and a sufficient number of sampling sites. The mrDMDcc algorithm can easily include new, emerging patterns in PM_2.5_ pollution and demographic information for determining future monitor placement. Finally, this framework may be applied to the existing EPA and PurpleAir networks to determine the optimal and equitable placement of new sensor locations.

## Conflict of Interest

The authors declare no conflicts of interest relevant to this study.

## Supporting information

Supporting Information S1

## Data Availability

The mrDMD algorithm and sensor network locations that support the findings of this study are openly available at the following URL/DOI: https://doi.org/10.5281/zenodo.7686016.
