# Supplementary material for: Data‐Driven Placement of PM2.5 Air Quality Sensors in the United States: An Approach to Target Urban Environmental Injustice
Source: Geohealth. 2023 Sep 13;7(9):e2023GH000834. doi: 10.1029/2023GH000834 (PMC10499371; doi:10.1029/2023GH000834)
Supplement: Supplementary file 1 — Supporting Information S1 [file GH2-7-e2023GH000834-s001.pdf]

Supplementary Materials for

Data-driven placement of $PM_{2.5}$ air quality sensors in the United States: An approach to target urban environmental injustice

[1]Makoto M. Kelp, [2]Timothy C. Fargiano, [3]Samuel Lin, [4]Tianjia Liu, [5]Jay R. Turner, [6]J. Nathan Kutz, and [7]Loretta J. Mickley

[1]Department of Earth and Planetary Sciences, Harvard University, Cambridge, MA 02138, USA
[2]Center for the Environment, Harvard University, Cambridge, MA 02138, USA
[3]Department of Computer Science, Harvard University, Cambridge, MA 02138, USA
[4]Department of Earth System Science, University of California, Irvine, Irvine, CA 92697, USA
[5]Department of Energy, Environmental and Chemical Engineering, Washington University, St. Louis, MO 63130, USA
[6]Department of Applied Mathematics, University of Washington, Seattle, WA 98195, USA
[7]John A. Paulson School of Engineering and Applied Sciences, Harvard University, Cambridge, MA 01238, USA

*Corresponding author. Email: mkelp@g.harvard.edu

This PDF file includes:

Figures S1 to S13

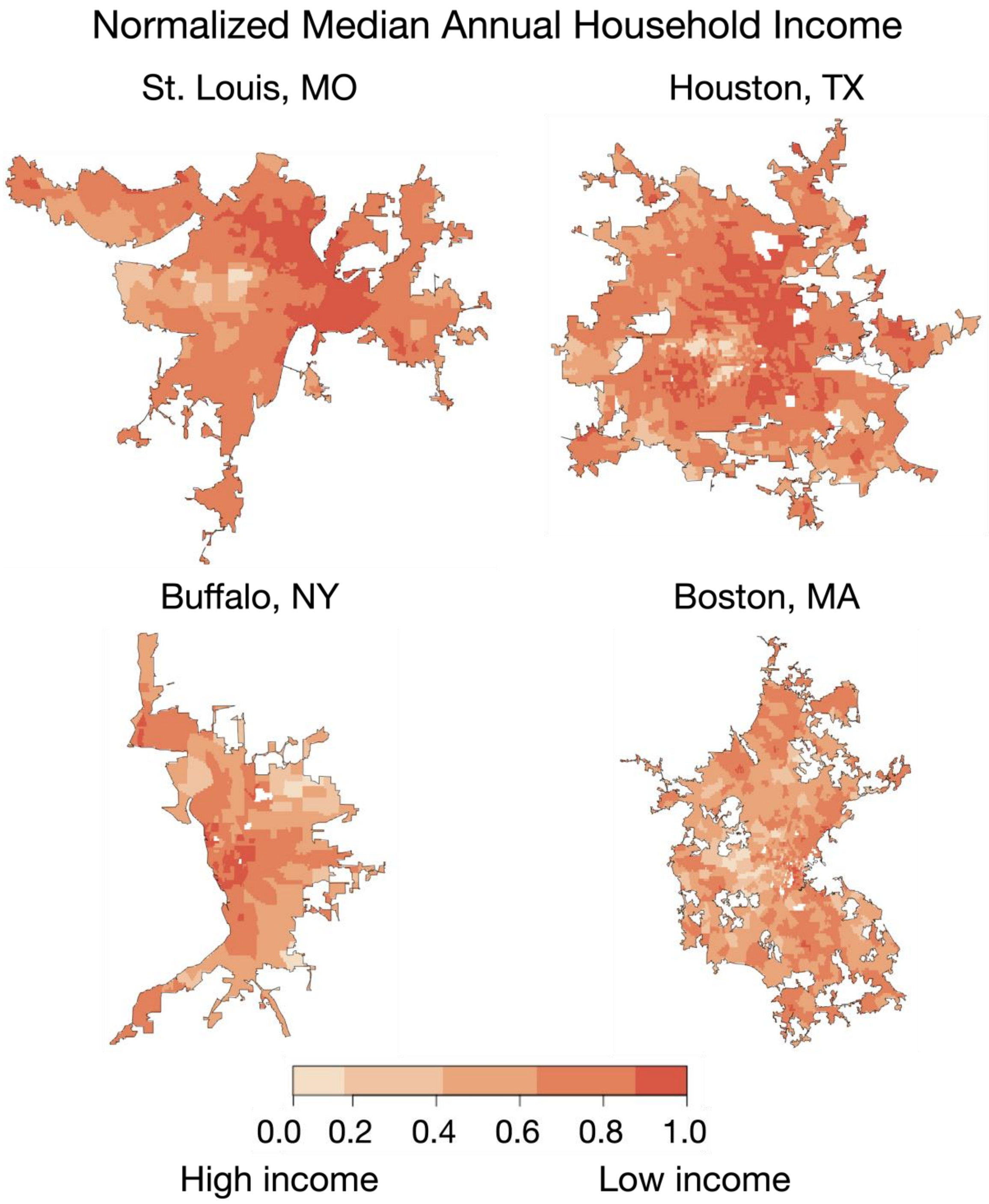

Figure S1. Maps of normalized annual median income for St. Louis, Houston, Buffalo, and Boston metropolitan areas. The median annual household income is obtained from the 2020 American Community Survey interpolated onto the centroids of the Di et al (2021). $PM_{2.5}$ dataset. The income landscape is normalized to the maximum median income across grid cells of an urban area ($250,000 for St. Louis, Houston, and Boston; $150,000 for Buffalo) then inverted such that 1.0 represents the lowest normalized median income and 0.0 represents the highest.

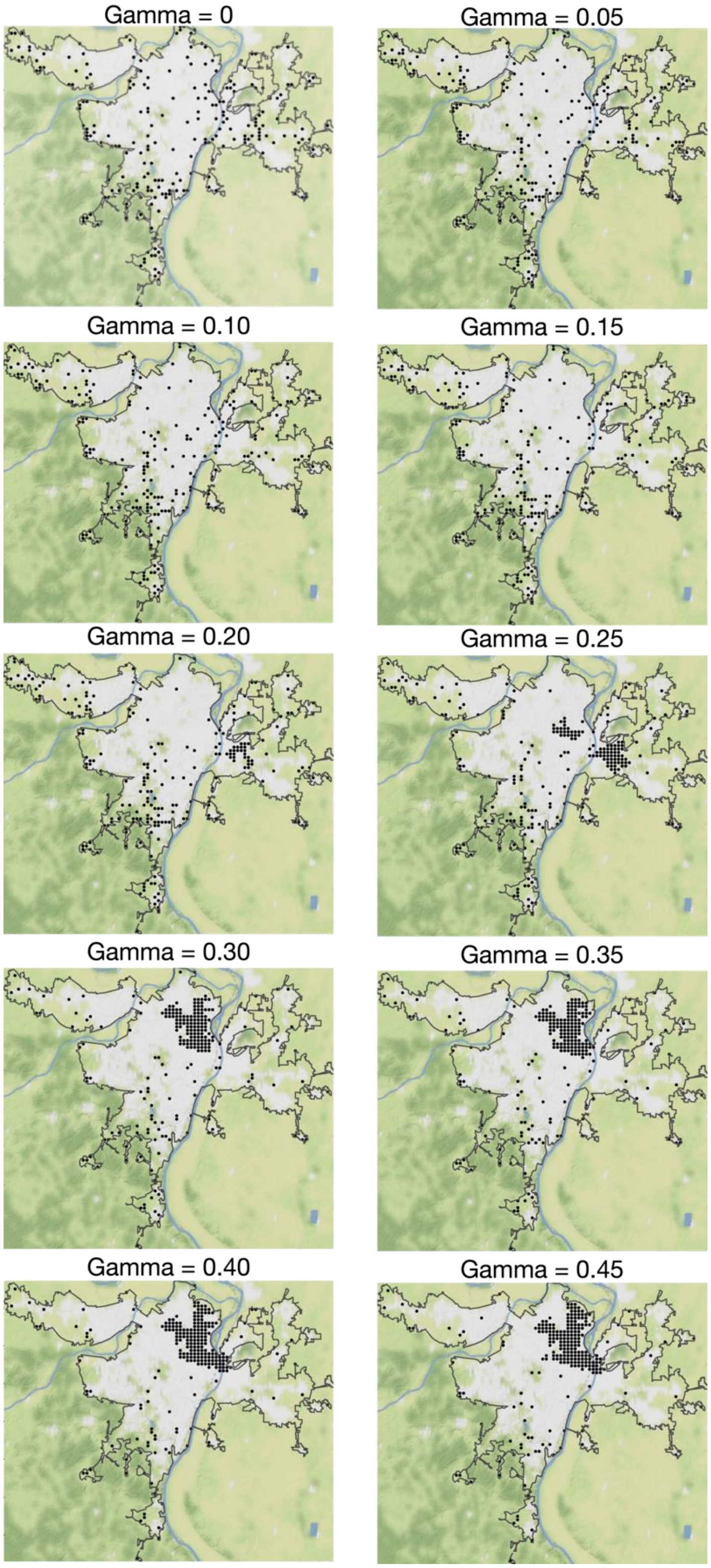

Figure S2. Effect of γ on sensor placement in St. Louis. The number of sensors is fixed at 250 for each value of γ. The γ value represents the forcing term that prioritizes the cost function (here being nonwhite grid cells) more than information from the $PM_{2.5}$ air pollution modes. All sensor locations are gridded onto the same 1 km x 1 km Di et al. (2021) grid.

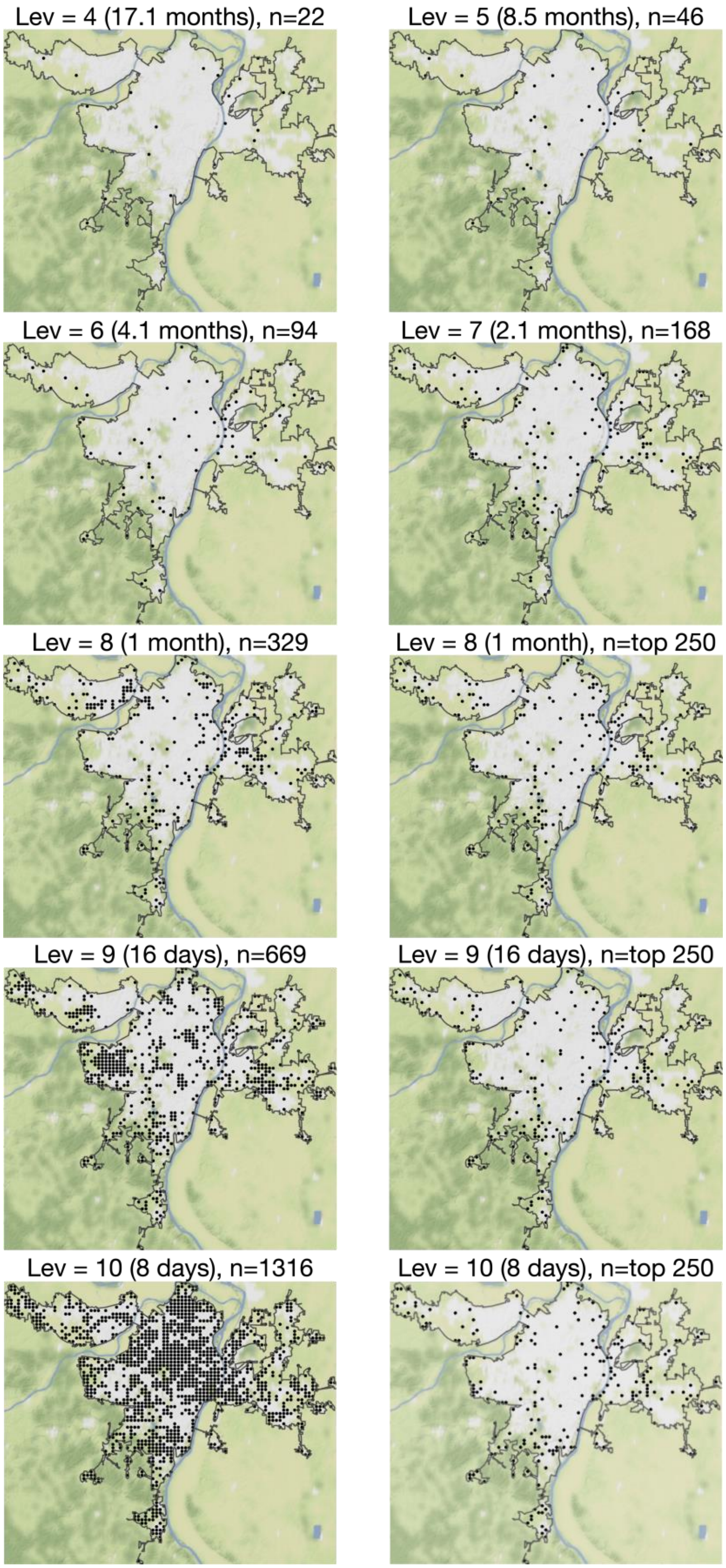

Lev = 4 (17.1 months), n=22
Lev = 5 (8.5 months), n=46
Lev = 6 (4.1 months), n=94
Lev = 7 (2.1 months), n=168
Lev = 8 (1 month), n=329
Lev = 8 (1 month), n=top 250
Lev = 9 (16 days), n=669
Lev = 9 (16 days), n=top 250
Lev = 10 (8 days), n=1316
Lev = 10 (8 days), n=top 250

Figure S3. Effect of higher temporal resolution modes on sensor placement distributions in St. Louis. The specified decomposition level and time window correspond to the modal information depicted in Figure S5. When fewer decomposition levels are incorporated into the mrDMD optimization, fewer sensors are required to capture pollution episodes, as there are generally fewer long-lived $PM_{2.5}$ modes. Conversely, including more decomposition levels introduces additional pollution episodes, necessitating a greater number of sensors to capture the complete spatiotemporal dynamics of $PM_{2.5}$. Incorporating weekly timescales (Lev = 10) would inundate the proposed sensor network. Instead, we select the 250 most variable sensors for our analysis. This choice enables the inclusion of weekly temporal dynamics while still maintaining a reasonable number of sensors for a major urban area in the United States.

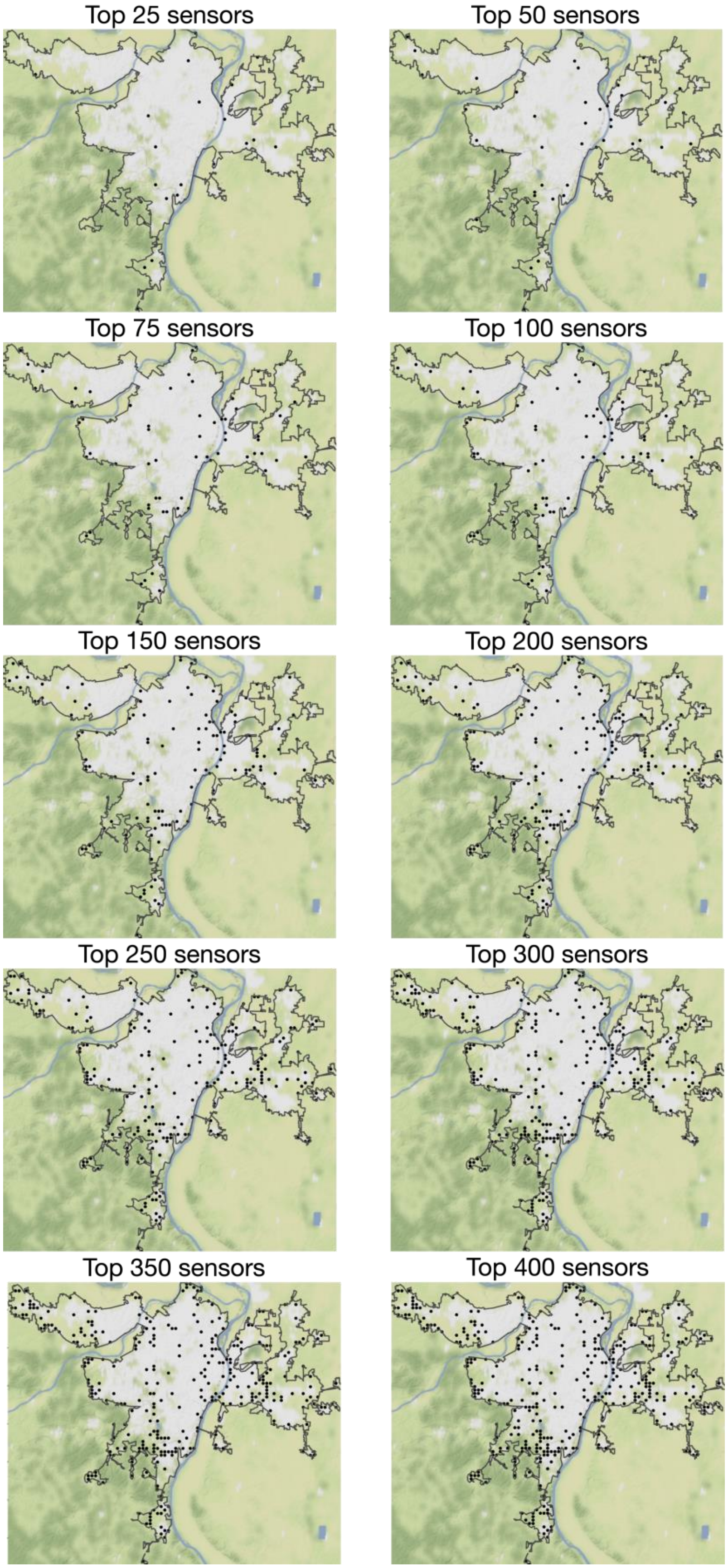
Top 25 sensors
Top 50 sensors
Top 75 sensors
Top 100 sensors
Top 150 sensors
Top 200 sensors
Top 250 sensors
Top 300 sensors
Top 350 sensors
Top 400 sensors

Figure S4. Varying the number of sensors in St. Louis. Distribution of sensor locations identified as optimal by the mrDMD algorithm. All sensor locations are gridded onto the same 1 km x 1 km Di et al. (2021) grid. Each panel shows the top number of sensors for a mrDMD decomposition using weekly time windows. Generally, under 100 sensors fails to capture many socioeconomic communities while more than 300 sensors start to observe overlapping spatial clustering which may be redundant.

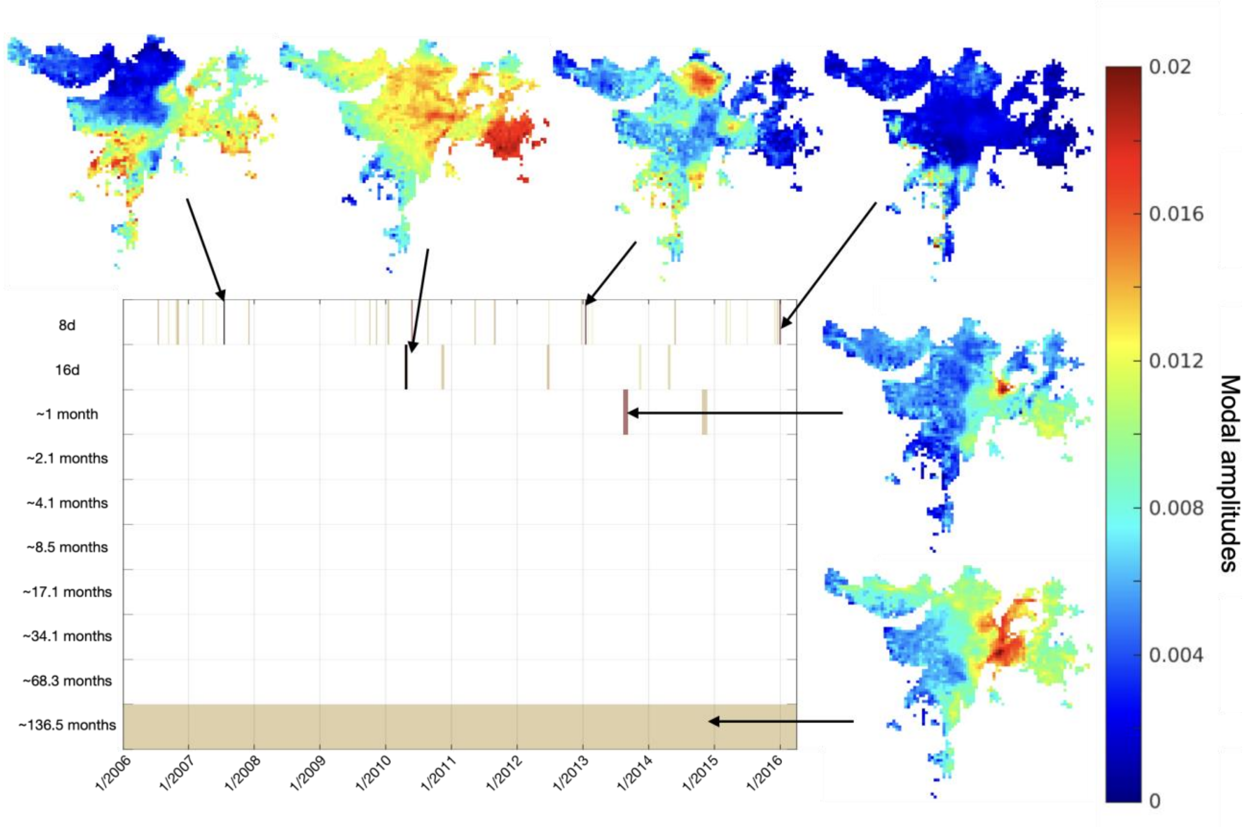

Figure S5. Maps of St. Louis mrDMD $PM_{2.5}$ modes. The mrDMD time window is for September 2005 to December 2016. The left axis expresses the decomposition level and its related time frequency, such that the bottom row corresponds to the average background mode of $PM_{2.5}$ over 4096 d (~136.5 months), with each successively higher row corresponding to pollution episodes lasting half the number of days of the row below. Colored boxes indicate a mrDMD mode that exhibits significant variability above the background mode; otherwise, the boxes are left blank. At each level of decomposition, the separation of the pollution episode from the background mode is deemed significant if its eigenvalues exceed a tolerance of 1. Here, we set this tolerance to a standard value of $1\ x\ 10^{-2}$. Darker shades of the colored boxes represent a relatively larger modal signal then the background mode. Modal maps of the background averages and examples of significant pollution episodes are shown in the margins. Arrows point to the time periods of the corresponding mrDMD modal maps. All mrDMD modes are on the same amplitude scale shown to the right.

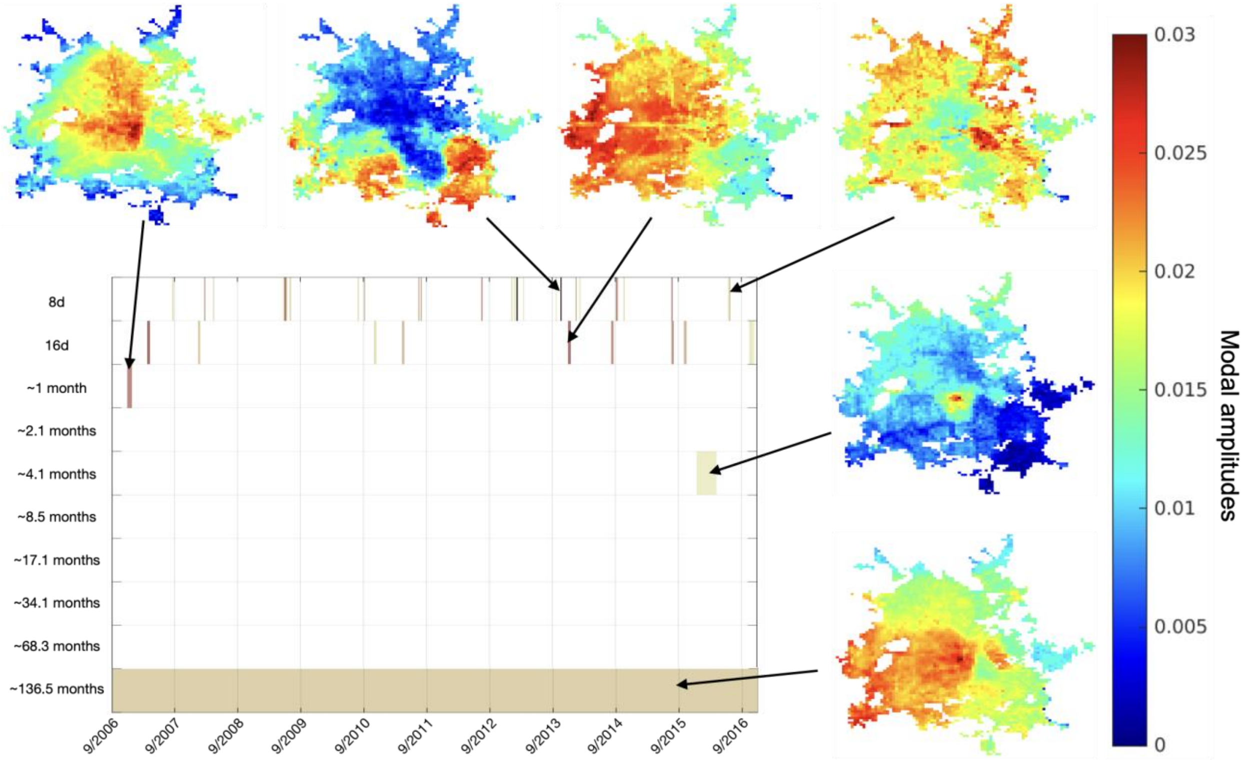

Figure S6. Maps of Houston mrDMD $PM_{2.5}$ modes. The mrDMD time window is for September 2005 to December 2016. The left axis expresses the decomposition level and its related time frequency, such that the bottom row corresponds to the average background mode of $PM_{2.5}$ over 4096 d (∼136.5 months), with each successively higher row corresponding to pollution episodes lasting half the number of days of the row below. Colored boxes indicate a mrDMD mode that exhibits significant variability above the background mode; otherwise, the boxes are left blank. At each level of decomposition, the separation of the pollution episode from the background mode is deemed significant if its eigenvalues exceed a tolerance of 1. Here, we set this tolerance to a standard value of $1\ x\ 10^{-2}$. Darker shades of the colored boxes represent a relatively larger modal signal then the background mode. Modal maps of the background averages and examples of significant pollution episodes are shown in the margins. Arrows point to the time periods of the corresponding mrDMD modal maps. All mrDMD modes are on the same amplitude scale shown to the right.

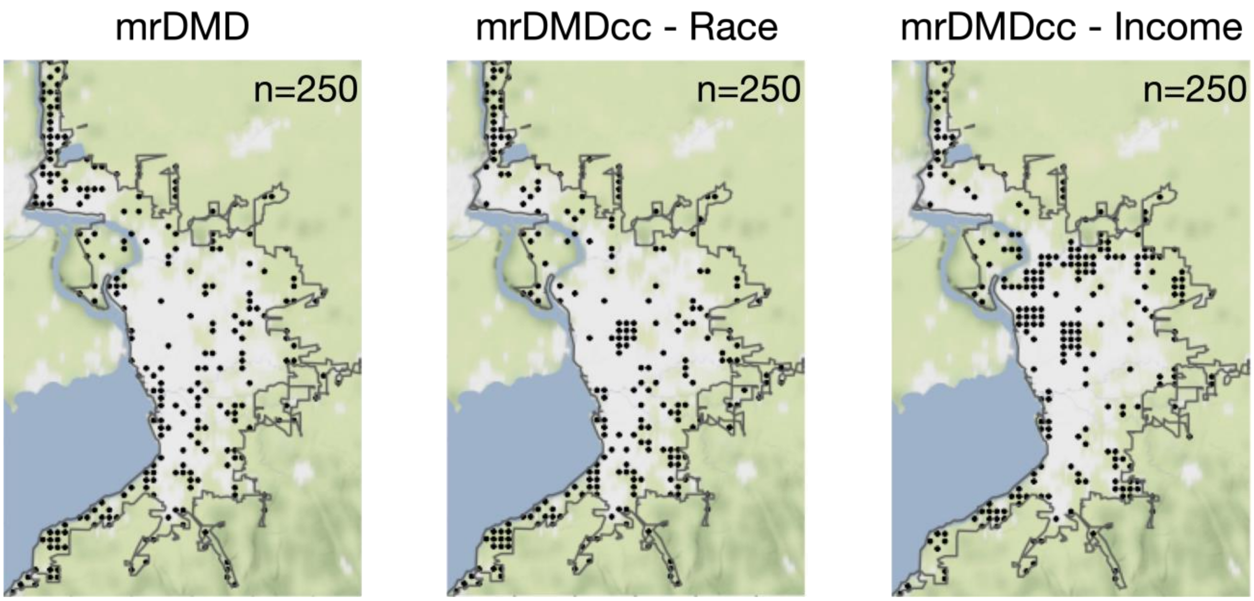

Figure S7. $PM_{2.5}$ sensor locations for Buffalo showing 250 sensors. The figure shows the distribution of sensor locations identified as optimal by the mrDMD algorithm, and those identified as optimal and equitable by the mrDMDcc using race and income metrics. All sensor locations are gridded onto the same 1 km x 1 km Di et al. grid. White areas of the sensor location maps represent the built environment, while the shades of green represent the natural vegetation colors of the area.

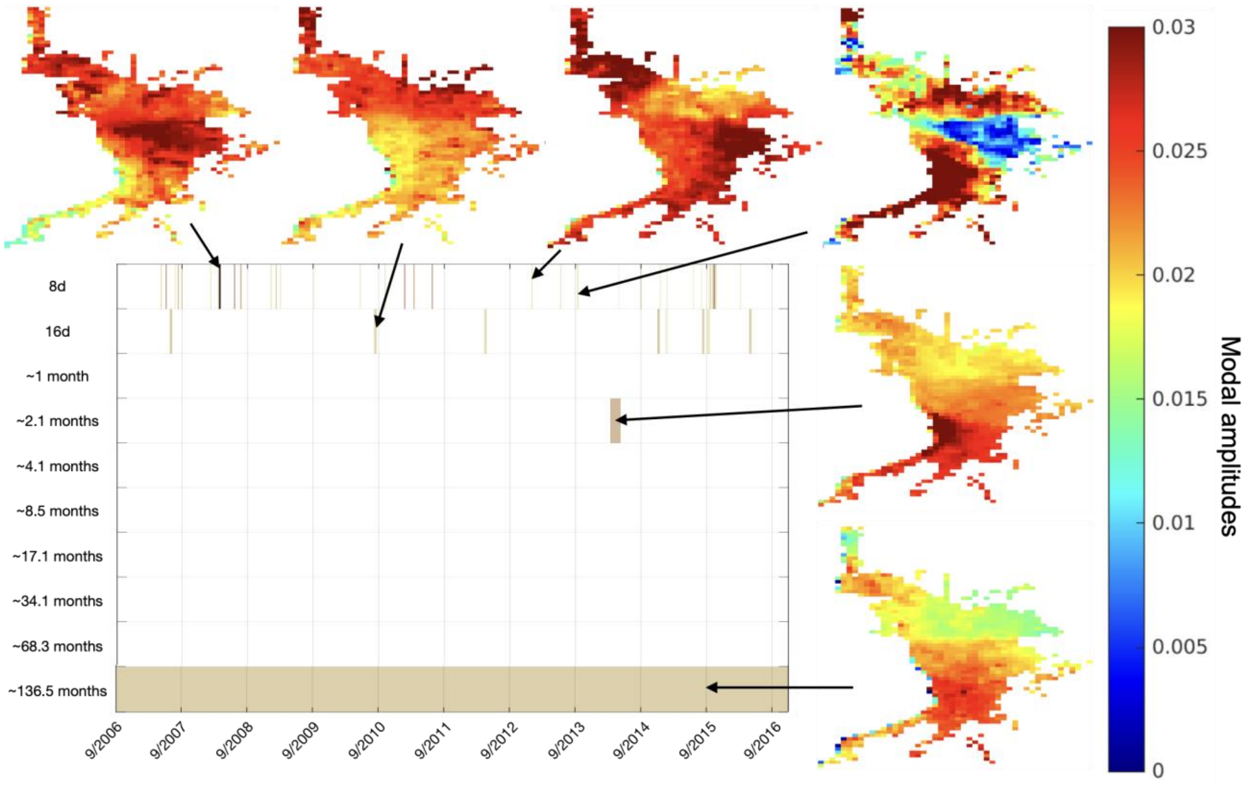

Figure S8. Maps of Buffalo mrDMD $PM_{2.5}$ modes. The mrDMD time window is for September 2005 to December 2016. The left axis expresses the decomposition level and its related time frequency, such that the bottom row corresponds to the average background mode of $PM_{2.5}$ over 4096 d (∼136.5 months), with each successively higher row corresponding to pollution episodes lasting half the number of days of the row below. Colored boxes indicate a mrDMD mode that exhibits significant variability above the background mode; otherwise, the boxes are left blank. At each level of decomposition, the separation of the pollution episode from the background mode is deemed significant if its eigenvalues exceed a tolerance of 1. Here, we set this tolerance to a standard value of $1\ x\ 10^{-2}$. Darker shades of the colored boxes represent a relatively larger modal signal then the background mode. Modal maps of the background averages and examples of significant pollution episodes are shown in the margins. Arrows point to the time periods of the corresponding mrDMD modal maps. All mrDMD modes are on the same amplitude scale shown to the right.

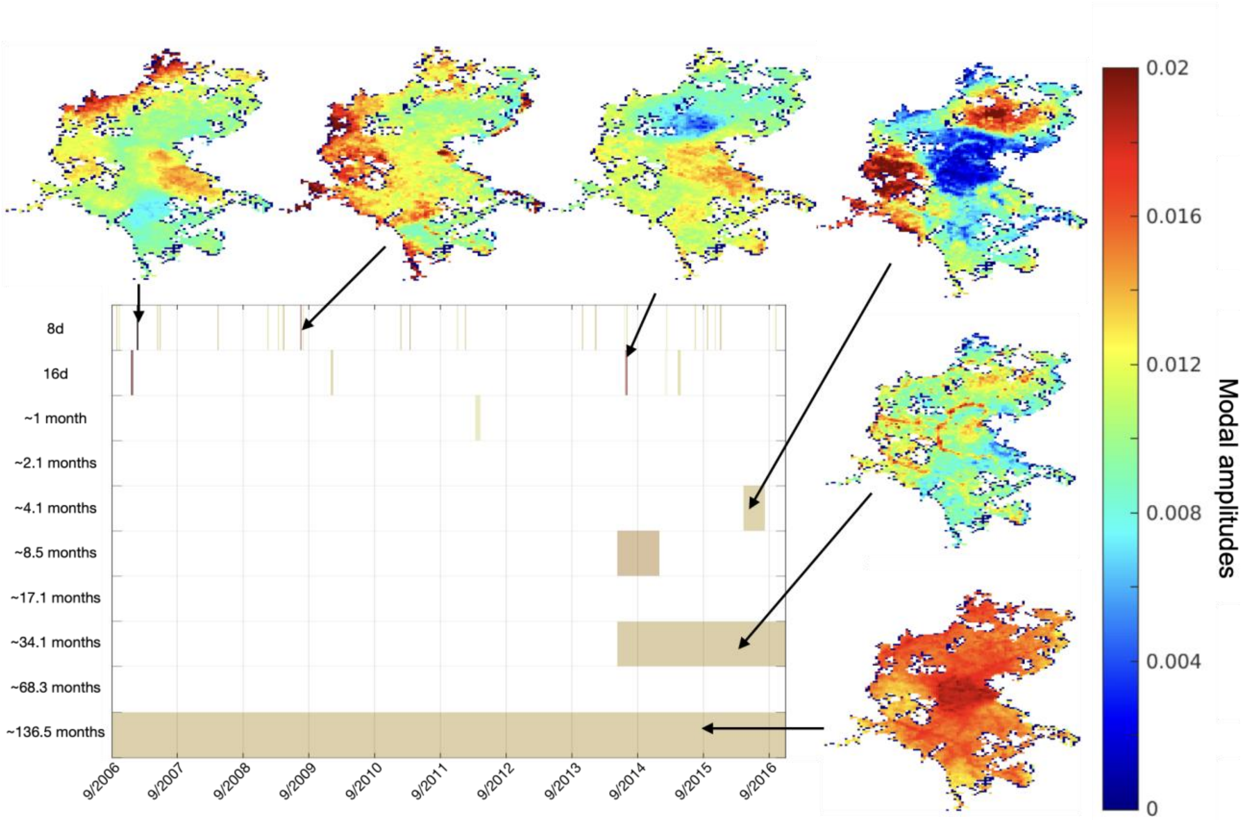

Figure S9. Maps of Boston mrDMD $PM_{2.5}$ modes. The mrDMD time window is for September 2005 to December 2016. The left axis expresses the decomposition level and its related time frequency, such that the bottom row corresponds to the average background mode of $PM_{2.5}$ over 4096 d (∼136.5 months), with each successively higher row corresponding to pollution episodes lasting half the number of days of the row below. Colored boxes indicate a mrDMD mode that exhibits significant variability above the background mode; otherwise, the boxes are left blank. At each level of decomposition, the separation of the pollution episode from the background mode is deemed significant if its eigenvalues exceed a tolerance of 1. Here, we set this tolerance to a standard value of $1\ x\ 10^{-2}$. Darker shades of the colored boxes represent a relatively larger modal signal then the background mode. Modal maps of the background averages and examples of significant pollution episodes are shown in the margins. Arrows point to the time periods of the corresponding mrDMD modal maps. All mrDMD modes are on the same amplitude scale shown to the right.

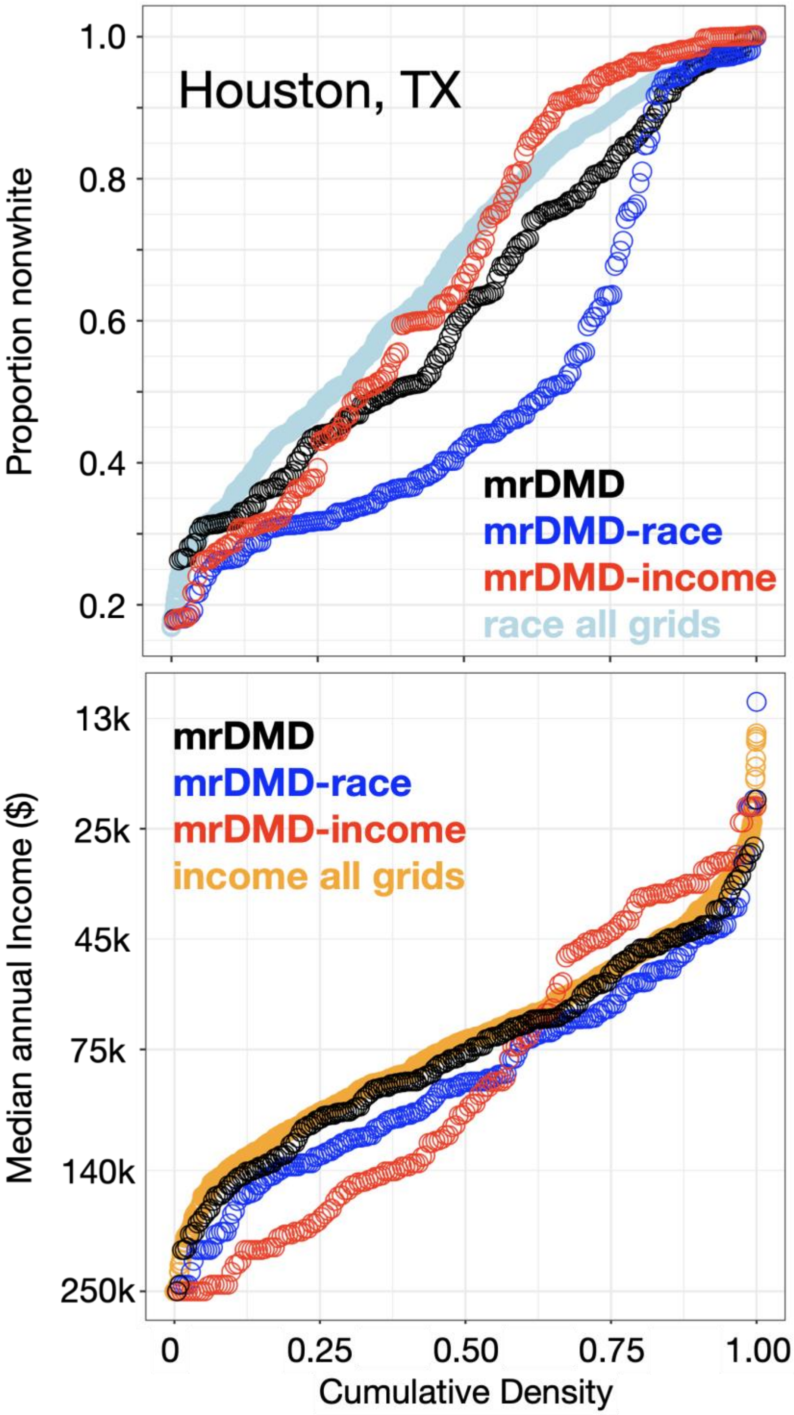

Figure S10. Cumulative frequency distributions for proportion of nonwhite locations and median annual income for the three different sensor network optimizations for Houston. Each point represents one sensor location out of the 250 designed for Houston. An additional set of points in each plot represents the distribution across racial composition (light blue) and income (orange) for a high-density, uniformly distributed sensor network across all 1 km^2 grid cells within the city bounds. The y-axis for median annual income has been reversed to make this panel consistent with the other panels, with the neighborhoods of greatest interest plotted at the high end of the distributions.

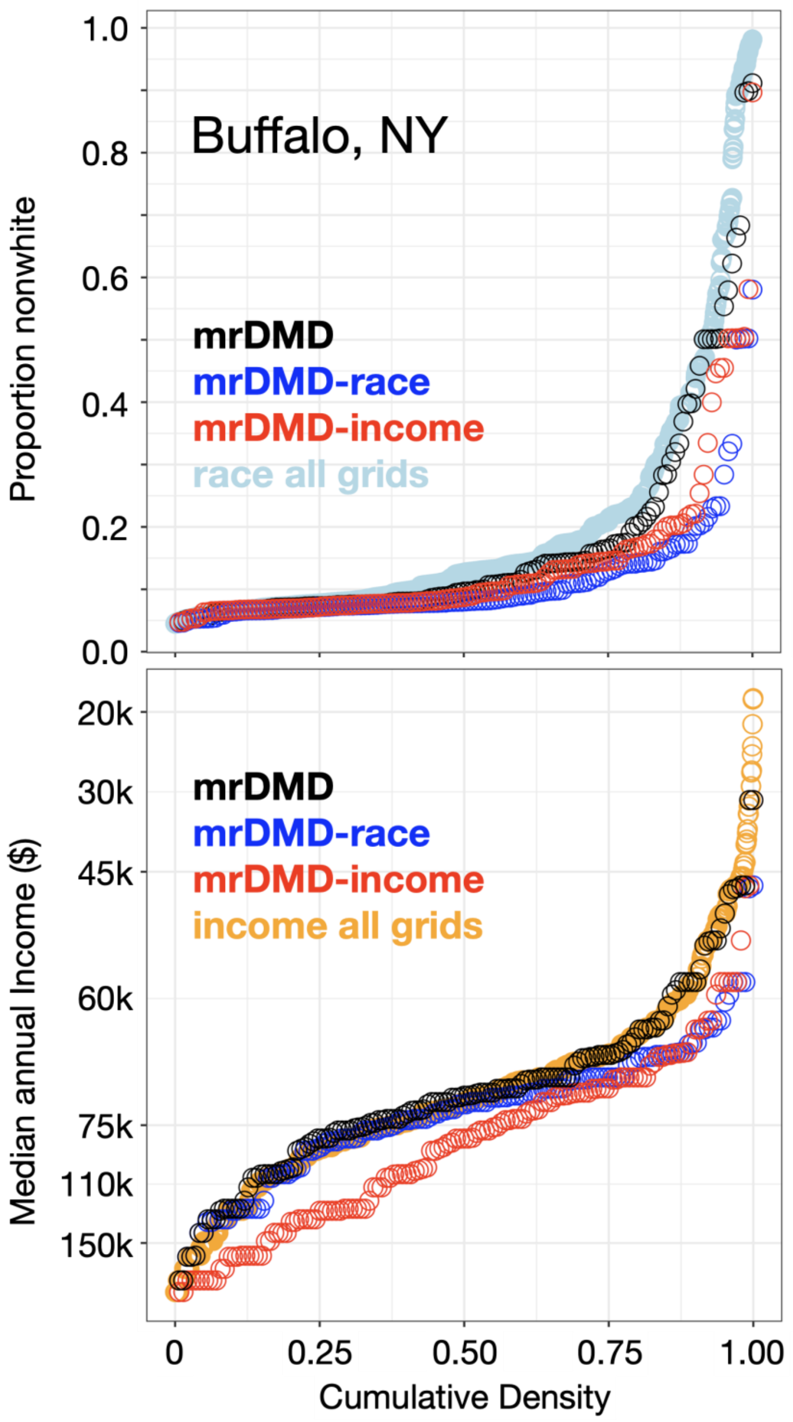

Figure S11. Cumulative frequency distributions for proportion of nonwhite locations and median annual income for the three different sensor network optimizations for Buffalo. Each point represents one sensor location out of the 150 designed for Buffalo. An additional set of points in each plot represents the distribution across racial composition (light blue) and income (orange) for a high-density, uniformly distributed sensor network across all 1 km^2 grid cells within the city bounds. The y-axis for median annual income has been reversed to make this panel consistent with the other panels, with the neighborhoods of greatest interest plotted at the high end of the distributions.

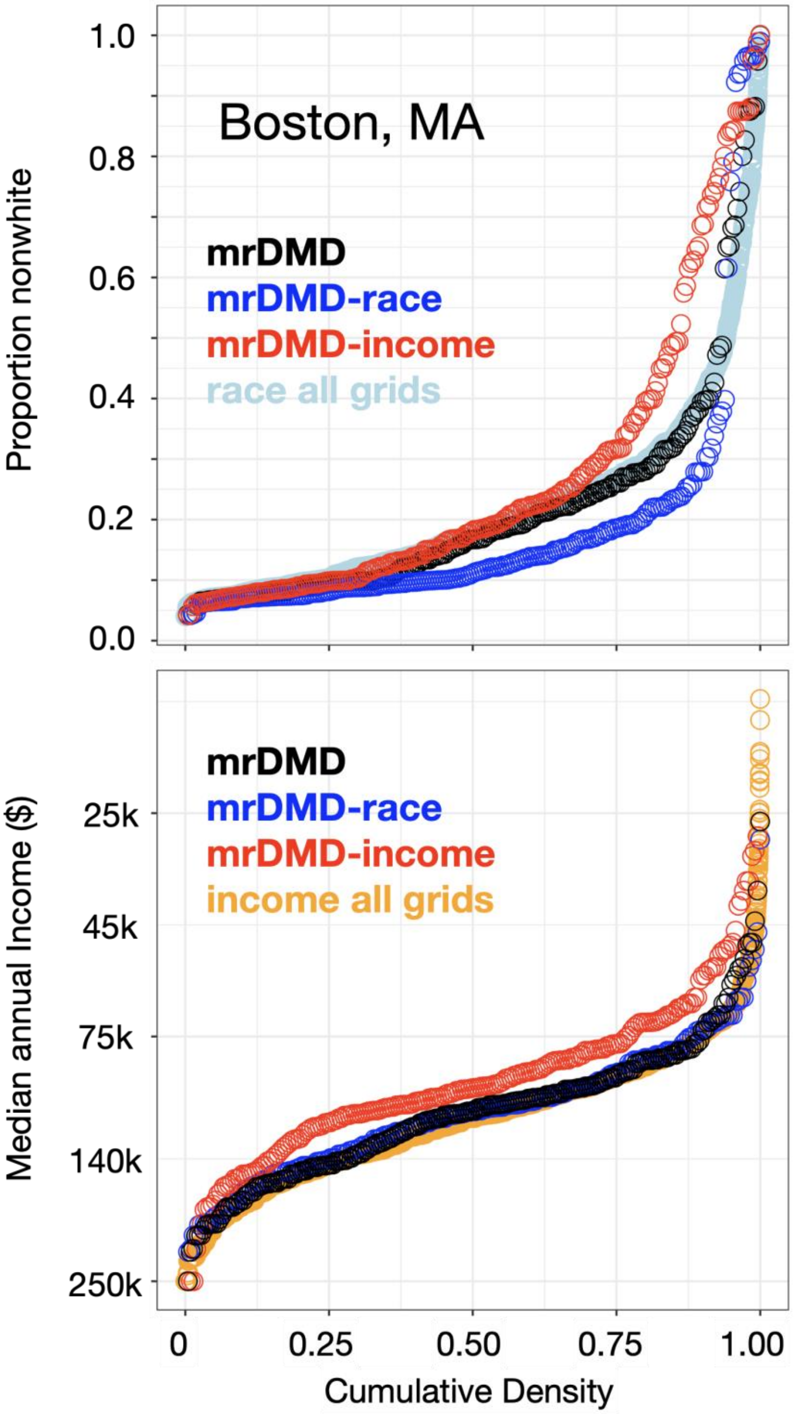

Figure S12. Cumulative frequency distributions for proportion of nonwhite locations and median annual income for the three different sensor network optimizations for Boston. Each point represents one sensor location out of the 250 designed for Boston. An additional set of points in each plot represents the distribution across racial composition (light blue) and income (orange) for a high-density, uniformly distributed sensor network across all 1 km^2 grid cells within the city bounds. The y-axis for median annual income has been reversed to make this panel consistent with the other panels, with the neighborhoods of greatest interest plotted at the high end of the distributions.

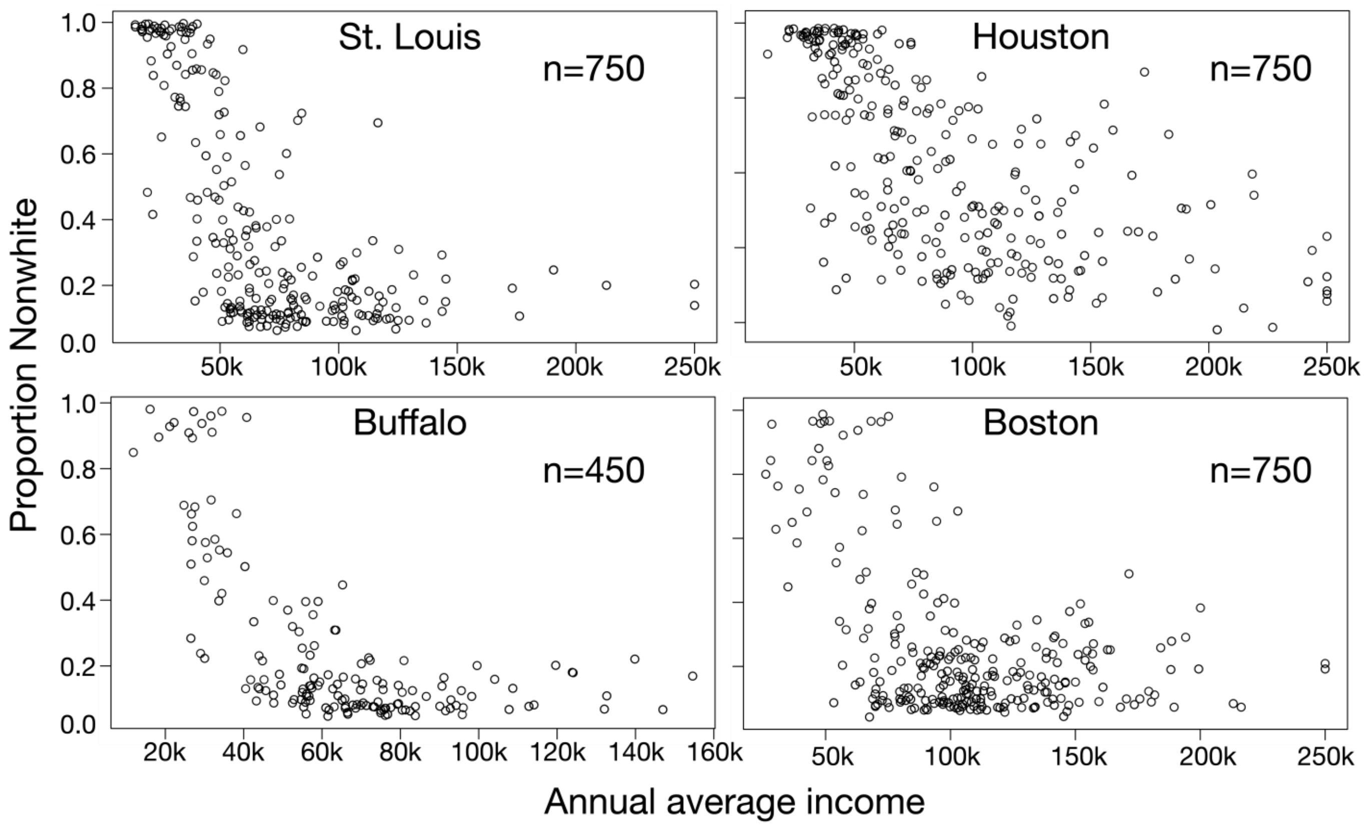

Figure S13. Scatterplot of annual average income ($USD) vs. proportion nonwhite for all sensor locations designed with the three sensor network optimizations for St. Louis, Houston, Buffalo, and Boston.
